# Toxoplasmosis Presenting as Nonhealing Cutaneous Ulcer

**DOI:** 10.1155/2020/8874800

**Published:** 2020-08-03

**Authors:** M. Adhikari, S. Dhakal, S. Bhattarai, U. Rai

**Affiliations:** Department of Pathology, BPKIHS, Nepal

## Abstract

**Introduction:**

Systemic manifestation of toxoplasmosis is commonly seen in immune-compromised individuals. Skin manifestations are seen commonly in conjunction with systemic features. Isolated cutaneous toxoplasmosis is extraordinarily rare in immunocompetent patients. *Case Description*. A 64-year-old female presented to the Dermatology Outpatient Department (OPD), with a nonhealing ulcer over dorsum of the left hand for one year. The patient did not have any systemic diseases. Serology tests were negative. An incisional biopsy of the lesion revealed dense inflammatory cell infiltrates comprising predominantly of plasma cells and lymphocytes, multinucleated giant cells, and focal abscess formation in the dermis. Periodic Acid Schiff (PAS) stain showed organisms in the dermis with morphological resemblance to tachyzoites of Toxoplasma gondii.

**Conclusion:**

Though rare, a possibility of primary cutaneous toxoplasmosis should always be considered and looked for, even in immunocompetent patients presenting with chronic nonhealing ulcers.

## 1. Introduction

Toxoplasmosis is a parasitic disease caused by the obligate intracellular coccidian protozoan Toxoplasma gondii, a tiny crescent-shaped parasite. Although cat is the definite host, humans can serve as an intermediate host [1]. Though systemic manifestation of toxoplasmosis is quite common, cutaneous involvement by it is rare. Most of the cutaneous cases of toxoplasmosis are associated with systemic involvement in an immunocompromised host. Isolated cutaneous toxoplasmosis is extraordinarily rare in immunocompetent patients [1, 2].

## 2. Case Report

**Figure 1:**
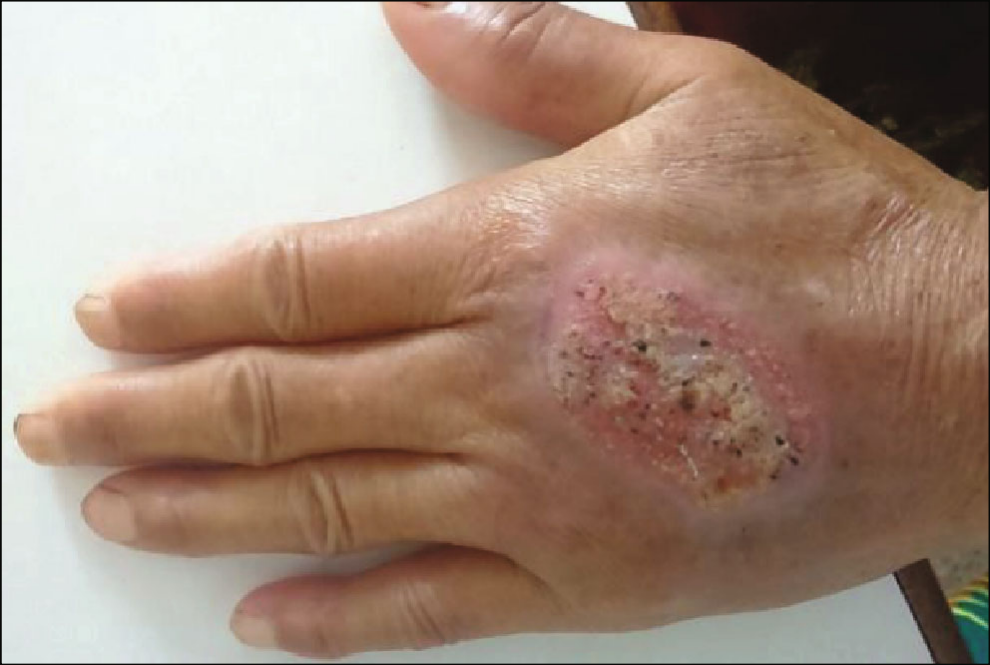
Ulcerated lesion in hand.

A 64-year-old female presented to the Dermatology Outpatient Department, BPKIHS, with complaints of a nonhealing ulcer over dorsum of the left hand for one year. There was no any history of diabetes, hypertension, and tuberculosis and no any history of trauma over the site. The lesion started as a small papule which over time increased in size and got ulcerated. On examination, there is crusted plaque with oozing pus mixed with blood with granulation tissue at the base of size 3 × 3 cm (Figure 1).

## 3. Histopathology

**Figure 2:**
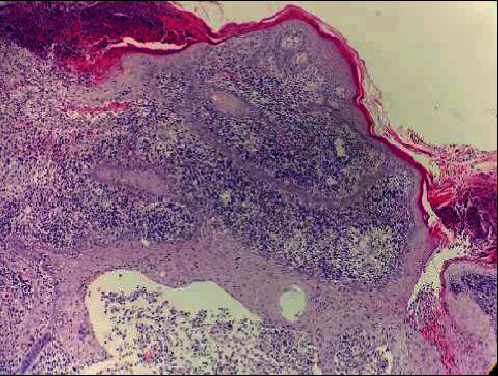
H&E section (100x magnification) revealing surface ulceration and crusting with dense inflammatory cell infiltrates in the dermis, at places forming pocket abscess.

An incisional biopsy of the lesion was sent for histological examination which showed epidermis lined by keratinized stratified squamous epithelium with parakeratosis, plasma crusting and focal neutrophilic infiltration, and pseudocarcinomatous hyperplasia. Dermis revealed suppurative to early plasma cell granulomas, along with eosinophils, macrophages, multinucleated giant cells, and focal abscess formation (Figure 2).

**Figure 3:**
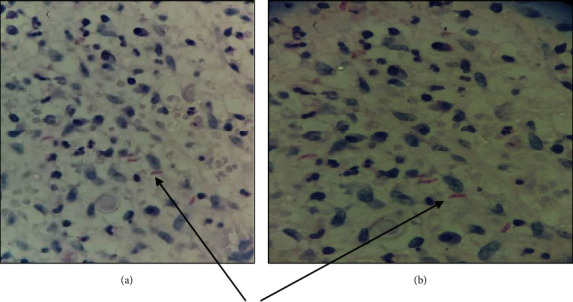
(a, b) Tachyzoites of Toxoplasma, highlighted by PAS stain (400x magnification).

Crescent-shaped organisms with a pointed anterior end and rounded posterior end were seen in the dermis with morphological resemblance to tachyzoites of Toxoplasma gondii which were positive for Periodic Acid Schiff (PAS) but negative for Silver Methenamine (SM) (Figures 3(a) and 3(b)).

## 4. Discussion

Pinkerton and Henderson were the first to describe cases of cutaneous toxoplasmosis in 1941 [3].

Cutaneous toxoplasmosis is usually found in patients with a compromised immune system, such as transplanted patients or patients having acquired immunodeficiency syndrome (AIDS) [1]. Cutaneous lesions are rarely observed in immune-competent patients [4].

Humans usually acquire this infection through ingestion of oocysts deposited in soil or litter pans of cats or by eating meat from chronically infected animals or through reactivation of a previous latent infection following HIV infection and transplacentally via tachyzoites [1]. Our patient gives the history of cat handling at home.

There are three infectious stages of *T. gondii*: the tachyzoites (in groups or clones), the bradyzoites (in tissue cysts), and the sporozoites (in oocysts).

The tachyzoite is often crescent-shaped, approximately 2 by 6 *μ*m, and with a pointed anterior (conoidal) end and a rounded posterior end. The nucleus is usually situated toward the central area of the cell and contains clumps of chromatin and a centrally located nucleolus [5].

During acute infection, rapidly multiplying “tachyzoites” occupy intracellular vacuoles; parasitized host cells are eventually destroyed. During chronic infection, the slowly multiplying organism “bradyzoites” store PAS-positive material and get tightly packed in “cysts.”

In our case, the stained tissue sections revealed crescentic structures that were PAS-positive.

Thus, a diagnosis of cutaneous toxoplasmosis was made, and the patient was put under ivermectin therapy, with subsequent healing of the lesion.

## 5. Conclusion

Though rare, a possibility of primary cutaneous toxoplasmosis should always be considered and looked for, even in immunocompetent patients presenting with chronic nonhealing ulcers.

## References

[1] Bajpai T., Bhatambare G., Nandedkar S., Malukani K. (2015). Unusual manifestation of isolated cutaneous toxoplasmosis in an immunocompetent patient. *Indian Journal of Dermatopathology and Diagnostic Dermatology*.

[2] Fong M. Y., Wong K. T., Rohela M. (2010). Unusual manifestation of cutaneous toxoplasmosis in a HIV-positive patient. *Tropical Biomedicine*.

[3] Leyva W. H., Santa Cruz D. J. (1986). Cutaneous toxoplasmosis. *Journal of the American Academy of Dermatology*.

[4] Marina S., Broshtilova V., Botev I. (2014). Cutaneous manifestations of toxoplasmosis: a case report. *Serbian Journal of Dermatology and Venereology*.

[5] Dubey J. P., Lindsay D. S., Speer C. A. (1998). Structures of Toxoplasma gondiiTachyzoites, bradyzoites, and sporozoites and biology and development of tissue cysts. *Clinical Microbiology Reviews*.

